# Hallucinogen-Like Action of the Novel Designer Drug 25I-NBOMe and Its Effect on Cortical Neurotransmitters in Rats

**DOI:** 10.1007/s12640-019-00033-x

**Published:** 2019-04-15

**Authors:** Monika Herian, Adam Wojtas, Katarzyna Kamińska, Paweł Świt, Anna Wach, Krystyna Gołembiowska

**Affiliations:** Department of Pharmacology, Institute of Pharmacology, Polish Academy of Sciences, 12 Smętna, 31-343 Kraków, Poland

**Keywords:** 25I-NBOMe, Neurotransmitter release, Dopamine, Serotonin, Glutamate, Head-twitch response

## Abstract

NBOMes are N-benzylmethoxy derivatives of the 2C family hallucinogens. 4-Iodo-2,5-dimethoxy-N-(2-methoxybenzyl)phenethylamine (25I-NBOMe) is one of the commonly used illicit drugs. It exhibits high binding affinity for 5-HT_2A/C_ and 5-HT_1A_ serotonin receptors. Activation of 5-HT_2A_ receptor induces head-twitch response (HTR) in rodents, a behavioral marker of hallucinogen effect in humans. There is not much data on neurochemical properties of NBOMes. Therefore, we aimed to investigate the effect of 25I-NBOMe on extracellular level of dopamine (DA), serotonin (5-HT), and glutamate (GLU) in the rat frontal cortex, tissue contents of monoamines, and hallucinogenic activity in rats. The extracellular levels of DA, 5-HT, and GLU were studied using microdialysis in freely moving animals. The tissue contents of DA, 5-HT and their metabolites 3,4-dihydroxyphenylacetic acid (DOPAC), homovanillic acid (HVA), and 5-hydroxyindoleacetic acid (5-HIAA) were determined in the rat frontal cortex. We also tested a drug-elicited HTR. 25I-NBOMe at doses 1, 3, and 10 mg/kg (sc) increased extracellular DA, 5-HT, and GLU levels, enhanced tissue content of 5-HT and 5-HIAA, but did not affect tissue level of DA and its metabolites. The compound exhibited an inverted *U*-shaped dose-response curve with respect to the effect on extracellular DA and 5-HT levels, but a *U*-shaped dose-response curve was observed for its effect on GLU release and HTR. The data from our study suggest that hallucinogenic activity of 25I-NBOMe seems to be related with the increase in extracellular GLU level-mediated via cortical 5-HT_2A_ receptors. The influence of 25I-NBOMe on 5-HT_2C_ and 5-HT_1A_ receptors may modulate its effect on neurotransmitters and HTR.

**Electronic supplementary material:**

The online version of this article (10.1007/s12640-019-00033-x) contains supplementary material, which is available to authorized users.

## Introduction

New psychoactive substances (NPS) are substitutes of well-known drugs with hallucinogenic effect on the central nervous system (CNS). The number of NPS that appear on the illicit drug market each year still remains high. According to EMCDDA (European Monitoring Centre for Drugs and Drug Addiction) by the end of 2017, above 670 new psychoactive substances have been identified and monitored. As for 2017, 51 new drugs have emerged, coming from many different classes of substances, e.g., synthetic cathinones, cannabinoids, or hallucinogens (EMCDDA 2018). These numbers show that although NPS have been marked as a target for legal restrictions in the USA and Europe, they still pose a threat for public health.

Hallucinogenic compounds have been known to mankind for centuries along with ethanol they might be one of the oldest known psychoactive substances (Nichols 2016; Schultes et al. 1998). They produce a variety of effects after administration, mostly revolving around visual and auditory hallucinations and mental distortions (Nichols 2004; UNODC 2013) and their use leads to a rapid development of tolerance, and cross-tolerance phenomenon (Abramson et al. 1956; Angrist et al. 1974; Wolbach et al. 1962). Hallucinogens can be divided into two main groups, namely indoleamines and phenylalkylamines, the latter can be also further broken into phenethylamines (such as mescaline, 2C-X compounds) and phenylisopropylamines (i.e., DOM) (Halberstadt 2017; Nichols 2012). Indoleamines include ergolines, rigid structural analogues related to LSD, and simple tryptamines, such as DMT or 5-methoxy-DMT (Nichols 2004, 2012; Halberstadt 2015). Phenylalkylamine hallucinogens are selective for 5-HT_2A_, 5-HT_2B_, and 5-HT_2C_ receptors, and some of these compounds display over 1000-fold selectivity for agonist-labeled 5-HT_2_ receptors versus 5-HT_1_ sites (Pierce and Peroutka 1989). The tryptamines, by contrast, bind non-selectively to 5-HT receptors and are substrates for the 5-HT transporter (SERT) (Nagai et al. 2007; Cozzi et al. 2009; Halberstadt and Geyer 2011). However, tryptamines are more potent at 5-HT_1A_ and 5-HT_2A_ receptors by several orders of magnitude as compared to SERT (Cozzi et al. 2009; Halberstadt and Geyer 2011; Nagai et al. 2007). The addition of *N*-benzyl group to phenethylamines potently increased their activity, and *N*-benzylphenethylamines are now a new class of hallucinogens (Braden et al. 2006).

Activation of 5-HT_2A_ receptor induces head-twitch response (HTR) in rodents which is a behavioral marker of hallucinogenic effect in humans. Serotonergic hallucinogens induce HTR in mice and rats also referred to as a wet dog shakes (WDS). The fact that DOI can provoke HTR in 5-HT_2C_ knockout mice but not in 5-HT_2A_ knockouts strongly suggests the 5-HT_2A_ receptor is responsible for mediating HTR (Canal et al. 2010). These findings indicate that 5-HT_2C_ activation inhibits HTR. On the other hand, the 5-HT_2A_ and 5-HT_1A_ receptors make positive and negative contribution, respectively, to the induction of HTR by tryptamine hallucinogens (Klein et al. 2018).

The enhanced release of glutamate is a common mechanism in the action of hallucinogens. In vivo microdialysis, experiments showed that systemic administration of DOI, 5-MeO-DIPT, and LSD significantly increased extracellular levels of glutamate in the rat frontal cortex (Muschamp et al. 2004; Noworyta-Sokołowska et al. 2016, 2019; Scruggs et al. 2003). The increase in glutamate elicited by intracortical DOI was blocked by the selective 5-HT_2A_ receptor antagonist MDL 100,907 (Scruggs et al. 2003).

Although most of behavioral effects of hallucinogens are due to activation of 5-HT_2A_ receptors, the higher doses of some hallucinogens may activate also 5-HT_2C_ receptors, which functionally oppose the effects of 5-HT_2A_ receptors. For instance, low doses of DOI increase locomotor activity in mice, whereas higher doses attenuate it (Halberstadt et al. 2009). The same phenomena can be observed in the mouse HTR (Fantegrossi et al. 2010).

It was hypothesized that the suppressant effect of tryptamine psychedelics and LSD on raphe cell firing results from stimulation of somatodendritic 5-HT_1A_ autoreceptors (Aghajanian and Haigler 1975). However, in subsequent studies, phenethylamine psychedelics which lack 5-HT_1A_ receptor agonist activity also suppressed raphe cells firing, but only when administered systematically but not directly into the raphe cells (Haigler and Aghajanian 1973). This suppressant effect by phenethylamine psychedelics is thought to occur through an indirect GABA-mediated mechanism (Martín-Ruiz et al. 2001).

A number of reports provide evidence for direct and indirect modulation of VTA dopaminergic neurons through activation of 5-HT_2A_ receptors (Cornea-Hébert et al. 1999). 5-HT_2A_ receptors may directly affect local release of DA, but some 5-HT_2A_ receptors can modulate VTA non-dopaminergic cells, perhaps GABAergic interneurons in VTA (Nocjar et al. 2002; Pehek et al. 2001).

NBOMes are N-benzylmethoxy derivatives of the 2C family of hallucinogens which are substituted phenethylamines. The presence of an *N*-benzyl group increased 5-HT_2A_ affinity of these drugs (Nichols 2016). The most commonly used NBOMe substances include 4-iodo-2,5-dimethoxy-N-(2-methoxybenzyl)phenethylamine, 4-bromo-2,5-dimethoxy-N-(2-methoxybenzyl)phenethylamine, and 4-chloro-2,5-dimethoxy-N-(2-methoxybenzyl)phenethylamine (25I-NBOMe, 25B-NBOMe and 25C-NBOMe, respectively) (Kyriakou et al. 2015). The NBOMes became available to drug users when they first appeared on the drug market in 2010, with 25I-NBOMe emerging as the earliest (Halberstadt 2017; Poklis et al. 2015; Zuba et al. 2013). NBOMes usually are sold on a blotter paper or in powder under various names: “Smiles,” “N-Bombs,” “Solaris,” “Cimbi,” or “25I,” “25B,” and “25C” (indicating their shortened chemical name). Decorated colorful papers infused with the drug are often distributed as lysergic acid diethylamide (LSD) due to a similar psychological and somatic effects (so-called “legal LSD”) (Suzuki et al. 2015). They are administered either nasally or orally (either by swallowing or sublingually) in submiligram doses (Poklis et al. 2015). The duration of action varies depending on the route of administration (Halberstadt and Geyer 2014). The effects of ingestion usually reflect the activation of serotonergic and adrenergic pathways and include severe visual and auditory hallucinations, agitation, aggressiveness, sweating, and psychotic/paranoid behavior (Al-Imam and Abdul Majeed 2017; Gee et al. 2016; Hill and Thomas 2011; Nikolaou et al. 2015). NBOMes have been associated with severe non-fatal toxicity and fatalities (Baumann et al. 2017; Halberstadt 2017; Kyriakou et al. 2015; Rose et al. 2013; Shanks et al. 2015; Walterscheid et al. 2014). Mild effects of NBOMe ingestion include tachycardia, hypertension, clonus, hallucinations, and panic attacks. In the case of heavy poisoning, seizures, renal failure, rhabdomyolysis, hyperthermia, and metabolic acidosis may occur (Andreasen et al. 2015; Kueppers and Cooke 2015). Similarly to other classes of hallucinogens, NBOMes act as 5-HT_2A_ receptor agonists and are relatively non-selective for 5-HT_2A_ vs. 5-HT_2C_ sites (Nichols et al. 2015). 25I-NBOMe exhibits high in vitro-binding affinity for 5-HT_1A_ (*K*_*i*_ = 1800 nM), 5-HT_2A_ (*K*_*i*_ = 0.6 nM), and 5-HT_2C_ receptors (*K*_*i*_ = 4.6 nM) (Rickli et al. 2015). 25I-NBOMe shows high affinity for adrenergic α_1A_ (*K*_*i*_ = 370 nM) and *α*_2A_ (*K*_*i*_ = 320 nM) receptors. Furthermore, binding affinity for *D*_1_, *D*_2_, and *D*_3_ receptors is 6700, 900, and 2100 nM, respectively (Rickli et al. 2015).

25I-NBOMe was potent in decreasing locomotor activity in mice (Gatch et al. 2017; Halberstadt 2017). Another study performed on mice showed no effects on locomotor activity after intraperitoneal administration of 25I-NBOMe (0.03–3 mg/kg). Instead, its subcutaneous administration at 0.1 mg/kg increased locomotor activity, while a dose of 3 mg/kg reduced this activity (Halberstadt 2017). Administration of NBOMe evoked HTR in C57BL/6J mice (Halberstadt and Geyer 2014) and in rats (Elmore et al. 2018). It is believed that NBOMes’ hallucinogenic properties result from the activation of the cortical 5-HT_2A_ receptors (Aghajanian and Marek 1997; Glennon et al. 1984; Sipes and Geyer 1995; Wing et al. 1990).

So far, there is not much neurochemical data about NBOMe compounds and their pharmacological properties. Therefore, the aim of this study was to investigate the effects of 25I-NBOMe on extracellular levels of dopamine (DA), serotonin (5-HT), and glutamate (GLU) in the frontal cortex, tissue content of neurotransmitters, and hallucinogenic activity of this compound in rats.

## Materials and Methods

### Animals

Male Wistar-Han rats (Charles River, Sulzfeld, Germany) weighting from 280 to 350 g were used in all performed experiments. Animals were initially acclimatized and housed (5 per cage) in environmentally controlled rooms under 12-h light/dark cycle (the light was switched on at 6 a.m.) at a temperature of 23 ± 1 °C and humidity of 55 ± 10%. Rats had free access to typical laboratory food and tap water (VRF 1, Special Diets Services, Witham, UK), enriched environment was not applied. The studies strictly conformed to European regulations for animal experimentation (EU Directive 2010/63/EU on the protection of animals used for scientific purposes). The experimental protocols were approved by the Local Ethics Commission for Experimentation on Animals (permit nos. 186 and 189/2017). This article does not contain any studies with human participants by any of the authors.

### Drugs and Reagents

2-(4-Iodo-2,5-dimethoxyphenyl)-N-(2-methoxybenzyl)-ethanamine (25I-NBOMe) was purchased from Cayman Chemical Company (Michigan, USA). All necessary chemicals for analysis with the use of high-performance liquid chromatography (HPLC) were obtained from Merck (Warszawa, Poland) and were of the highest purity. O-phthalaldehyde (OPA) obtained from Sigma-Aldrich was used for derivatization of glutamate to electroactive compound.

### Drug Administration

During the experiment animals received subcutaneous (sc) single injections of 25I-NBOMe dissolved in 0.9% NaCl at four doses of 0.3, 1, 3, and 10 mg/kg. The control group was administered 0.9% NaCl solution in the same way.

### Brain Microdialysis

Ketamine and xylazine solutions at doses of 75 and 10 mg/kg, respectively, were used to anesthetize animals. In the next step, animals were placed in the stereotaxic device (David Kopf Instruments, Tujunga, USA). The skin was peeled away from the skull, holes were drilled in the bone, and microdialysis probes (MAB 4.15.3Cu, AgnTho’s AB, Lindingö, Sweden) were implanted into the rat frontal cortex (FCx) using the following coordinates (mm): AP, + 2.7; L, + 0.8; V, − 6.5, from the dura (Paxinos and Watson 1998). The number of animals for each treatment group was six. On the next day, probe inlets were connected to a syringe pump (BAS, West Lafayette, IN, USA), which delivered artificial cerebrospinal fluid composed of (mM): 147, NaCl; 2.7, KCl; 1.0, MgCl_2_; 1.2, CaCl_2_, pH 7.4 at a flow rate of 2 μL/min. After a 2-h washout period, five basal dialysate samples were collected every 20 min, then animals were injected subcutaneously with 25I-NBOMe as indicated in the figure captions and fraction collection continued for 240 min. At the end of the experiment, the rats were sacrificed and their brains were histologically verified for the proper probe placement.

### Extracellular Concentration of DA, 5-HT, and Glutamate

The DA and 5-HT concentrations in dialysate fractions were analyzed by HPLC with electrochemical detection. Chromatography was performed using an Ultimate 3000 System (Dionex, Sunnyvale, CA, USA), electrochemical detector Coulochem III (model 5300; ESA, Chelmsford, MA, USA) with a 5020 guard cell, a 5040 amperometric cell, and a Hypersil Gold C18 analytical column (3 μm, 100 × 3 mm; Thermo Fischer Scientific, Waltham, MA, USA). The mobile phase was composed of 0.1 M potassium phosphate buffer adjusted to pH 3.8, 0.5 mM Na_2_EDTA, 100 mg/L 1-octanesulfonic acid sodium salt, and 2% methanol. The flow rate during analysis was set at 0.6 mL/min. The applied potential of a guard cell was 600 mV, while that of amperometric cell was 300 mV with a sensitivity set at 10 nA/V. The chromatographic data were processed by Chromeleon v. 6.80 (Dionex) software package run on a personal computer. The limit of detection of DA and 5-HT in dialysates was 0.002 pg/10 μL for DA and 0.01 pg/10 μL for 5-HT.

Glutamate levels in the extracellular fluids were measured electrochemically after derivatization with OPA/sulfite reagent to form isoindole-sulfonate derivative (Rowley et al. 1995). Chromatography was performed using an Ultimate 3000 pump (Dionex), LC-4B amperometric detector with a cross-flow detector cell (BAS), and a HR-80 column (3 μm, 80 × 4.6 mm; ESA Inc., Chelmsford, MA, USA). The mobile phase consisted of 100 mM monosodium orthophosphate at pH 4.6 and 4% methanol. The flow rate was 1 mL/min, and the applied potential of a 3-mm glassy carbon electrode was set at + 600 mV at a sensitivity of 5 nA/V. Glutamate-derivative peak was compared with the respective standard, and the data were processed using Chromax 2005 (Pol-Lab, Warszawa, Poland) software on a personal computer. The limit of detection of glutamate in dialysates was 0.03 ng/10 μL.

### The Measurement of Tissue Content of DA, 5-HT, and their Metabolites

Animals were sacrificed by decapitation 45 min after subcutaneous drug administration. Brains were removed and the frontal cortices were dissected in anatomical borders. The tissue levels of DA, 5-HT, 3,4-dihydroxyphenylacetic acid (DOPAC), homovanillic acid (HVA), and 5-hydroxyindoleacetic acid (5-HIAA) were measured using HPLC with electrochemical detection. Tissue samples of frontal cortices were homogenized in an ice-cold 0.1 M HClO_4_, and were centrifuged at 10,000×*g* for 10 min at 4 °C). The obtained supernatants were filtered through 0.22 μm Ultrafree Centrifugal Filters (Merck Millipore Ltd., Ireland) and 3–5 μL of samples were injected into an HPLC system. The chromatographic system consisted of an UltiMate 3000 pump (Thermo Scientific, USA), an LC-4C amperometric detector with a cross-flow detector cell (BAS, IN, USA), and a HR-80 analytical column (3 μm, 80 × 4.6 mm, ESA, Inc., USA). The mobile phase consisted of 0.1 M KH_2_PO_4_, 0.5 mM EDTA, 95 mg/L sodium 1-octanesulfonate, and 4% methanol, adjusted to pH = 3.8 with 85% H_3_PO_4_. The flow rate was 1 mL/min. The potential of a 3-mm glassy carbon electrode was set at + 700 mV with sensitivity of 5 nA/V. The temperature of the column was maintained at 30 °C. The obtained data were collected and processed by Chromax 2007 software (Pol-Lab, Warszawa, Poland).

### Head-Twitch Test

Head-twitch test was carried out during microdialysis experiment. Immediately after 25I-NBOMe administration, animals placed in microdialysis cylinders were observed by experimenters and the number of head twitches was counted for a period of 240 min.

### Data Analysis

Drug effects on DA, 5-HT, and glutamate release in the frontal cortex were analyzed with repeated measures ANOVA followed by Tukey’s post hoc test. All obtained data were presented as a percent of the basal level assumed to be 100%. The tissue content of neurotransmitters and HTR were tested using one-way ANOVA followed by Tukey’s post hoc test. The differences were considered significant if *P* value was smaller than 0.05. All statistical analyses were carried out using STATISTICA v.10 StatSoft Inc. 1984–2011 (San Francisco, CA, USA).

## Results

### The Effect of 25I-NBOMe Administration on the Extracellular Levels of DA, 5-HT, and Glutamate in the Rat Frontal Cortex

**Fig. 1:**
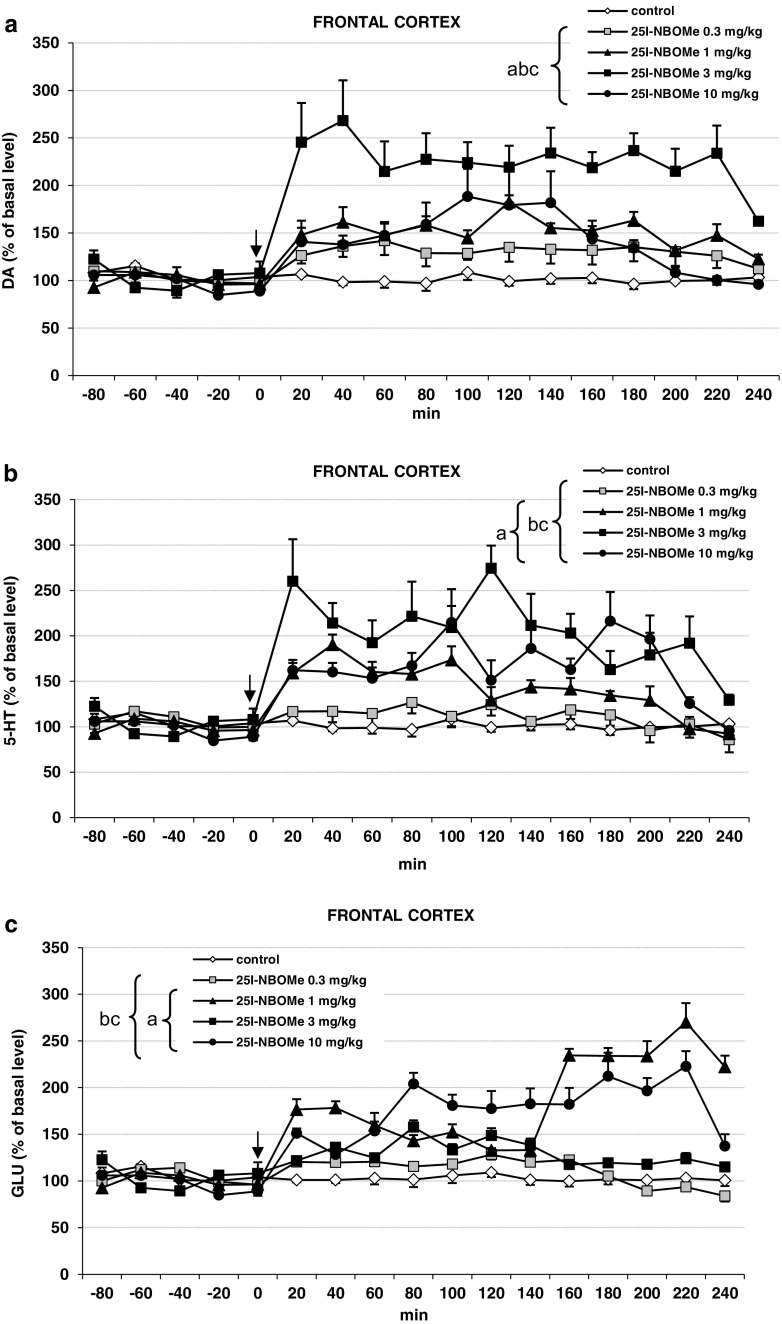
The effects of N-(2-methoxybenzyl)-2,5-dimethoxy-4-iodophenethylamine (25I-NBOMe) on extracellular levels of dopamine (DA), serotonin (5-HT), and glutamate (GLU) measured in the rat frontal cortex. Panels **a**, **b,** and **c** show each time-courses. Values are the mean ± standard error of the mean (SEM) (*n* = 6 per experimental groups). The time of drug injection is indicated with an arrow. The basal extracellular levels of DA were 2.22 ± 0.21 (*n* = 18); for 5-HT, 0.88 ± 0.07 (*n* = 18) in pg/10 μL: for GLU, 2.84 ± 0.32 (*n* = 18) in ng/10 μL; “a” *P* < 0.01 vs. control group; “b” and “c” *P* < 0.01 show significant difference vs. 3 and 0.3 mg/kg 25I-NBOMe, respectively (repeated measures ANOVA and Tukey’s post hoc test)

25I-NBOMe significantly (*P* < 0.0002) increased extracellular DA levels in the rat frontal cortex. The dose of 3 mg/kg was the most potent, the dose of 0.3 mg/kg was the least active, while the effect of 1 and 10 mg/kg doses was similar and weaker in comparison to the effect of a dose of 3 mg/kg (Fig. 1a). Repeated measures ANOVA showed a significant effect of treatment groups (*F*_4,25_ = 440, *P* < 0.0001), sampling period (*F*_11,275_ = 27, *P* < 0.0001), and the interaction between treatment groups and sampling period (*F*_44,275_ = 9.6, *P* < 0.0001).

The middle 25I-NBOMe dose of 3 mg/kg induced the strongest increase in the extracellular 5-HT level in the frontal cortex (Fig. 1b). The effect of 1 and 10 mg/kg was weaker but significant in comparison with control group while the dose of 0.3 mg/kg was inactive (Fig. 1b). Repeated measures ANOVA showed a significant effect of treatment groups (*F*_4,25_ = 659, *P* < 0.0001), sampling period (*F*_11,103_ = 96, *P* < 0.0001), and the interaction between treatment groups (*F*_44,275_ = 38, *P* < 0.0001).

The extracellular glutamate level was not changed by the dose of 0.3 mg/kg, but was increased more potently by 1 and 10 mg/kg doses than by the dose of 3 mg/kg (Fig. 1c). Repeated measures ANOVA showed a significant effect of treatment groups (*F*_4,25_ = 174, *P* < 0.0001), sampling period (*F*_11,275_ = 30, *P* < 0.0001), and the interaction between treatment groups (*F*_44,275_ = 41, *P* < 0.0001).

**Fig. 2:**
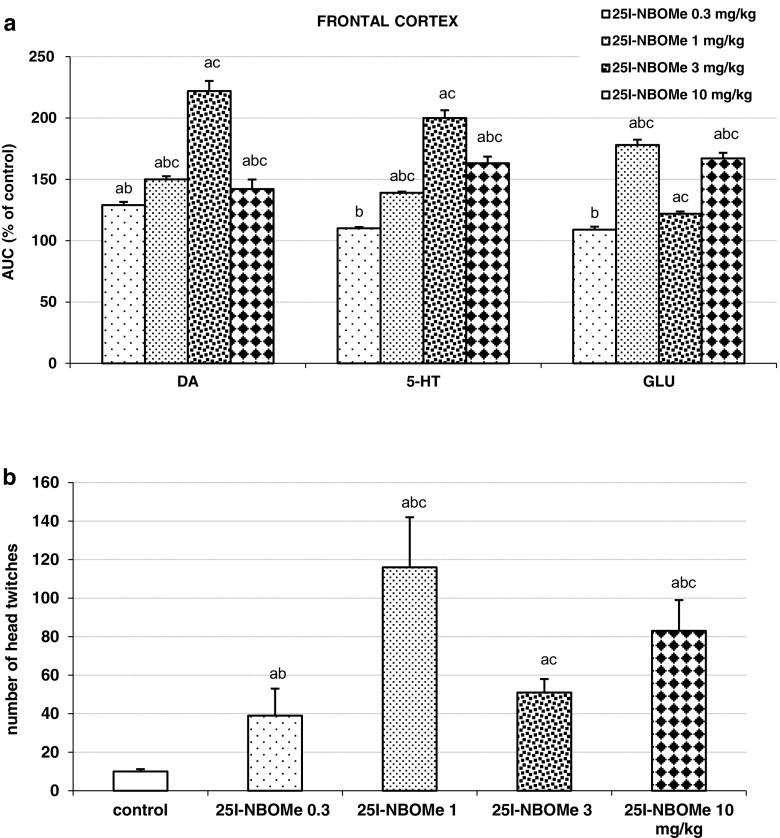
The effects of 25I-NBOMe on extracellular levels of DA, 5-HT, and GLU measured in the rat frontal cortex and head-twitch response induced by the drug. Panel **a** shows the total effects expressed as an area under the curve (AUC) of the percent of each basal level. Panel **b** shows the number of head twitches counted for 240 min starting immediately after injection. Values are the mean ± standard error of the mean (SEM) (*n* = 6 per experimental groups). “a” *P* < 0.01 vs. control group; “b” and “c” *P* < 0.01 show significant difference vs. 3 and 0.3 mg/kg 25I-NBOMe, respectively (one-way ANOVA and Tukey’s post hoc test)

The total effects expressed as AUC shown in Fig. 2a reflect the responses to 25I-NBOMe with respect to cortical DA, 5-HT, and glutamate release presented as time-course curves. The total effect of a 25I-NBOMe middle dose of 3 mg/kg was the most potent on DA and 5-HT release, whereas the dose of 0.3 mg/kg slightly but significantly increased DA release, but did not affect 5-HT release. Glutamate release was nearly equally increased by doses of 1 and 10 mg/kg, the dose of 3 mg/kg exerted the weakest but significant effect while the dose of 0.3 mg/kg was inactive in respect of glutamate release.

### The Effect of 25I-NBOMe on Head Twitches in Rats

25I-NBOMe induced head twitches in rats, which were observed immediately after the administration. The middle dose of 3 mg/kg produced a weak effect, while the impact of the lowest dose of 0.3 mg/kg was significant but less potent in comparison to the middle dose. The response of 1 and 10 mg/kg doses was significantly stronger in comparison to the two lower doses (Fig. 2b).

### The Effect of 25I-NBOMe on the Contents of DA, 5-HT, and their Metabolites in the Rat Frontal Cortex

**Fig. 3:**
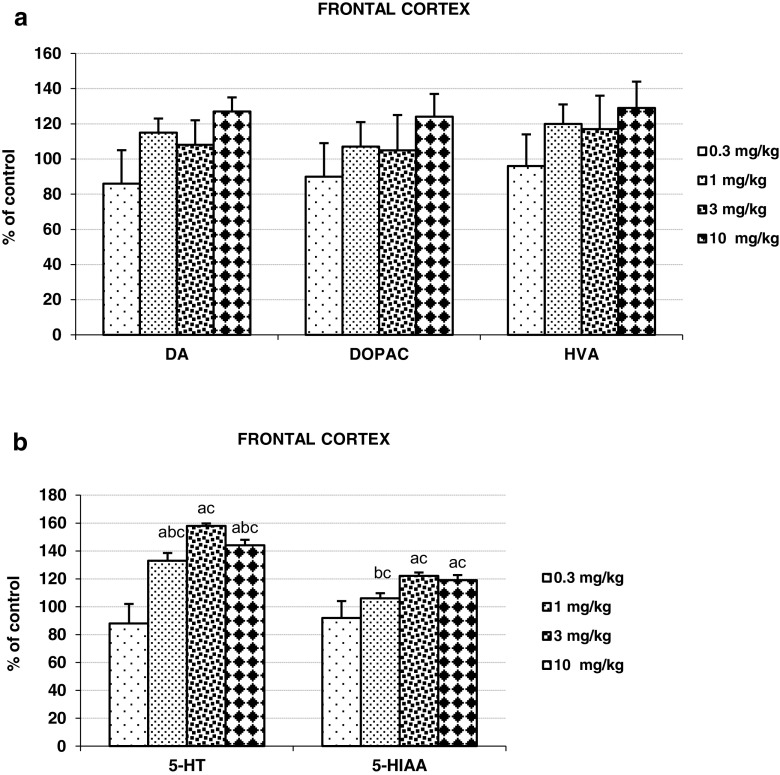
The effects of 25I-NBOMe on tissue contents of DA, DOPAC, HVA, 5-HT, and 5-HIAA in the rat frontal cortex measured 45 min after administration of the drug. Panels **a** and **b** show each percent change in comparison to control group and calculated from the absolute numbers given in nanograms/milligrams of tissue presented in supplementary materials. Values are the mean ± standard error of the mean (SEM) (*n* = 6 per experimental groups). “a” *P* < 0.01 vs. control group; “b” and “c” *P* < 0.01 show significant difference vs. 3 and 0.3 mg/kg 25I-NBOMe, respectively (one-way ANOVA and Tukey’s post hoc test)

25I-NBOMe did not affect significantly DA, DOPAC, and HVA tissue contents in the frontal cortex (Fig. 3a). However, the contents of 5-HT and 5-HIAA were increased in an inverted *U*-shaped manner with the strongest effect of the 3 mg/kg dose (Fig. 3b).

## Discussion

25I-NBOMe enhanced extracellular level of DA, 5-HT, and glutamate in the rat frontal cortex (Fig. 1a, b, c). It also increased cortical 5-HT and 5-HIAA tissue content (Fig. 3a, b). In addition, 25I-NBOMe produced hallucinogenic activity measured as head shake episodes (Fig. 2b).

The changes in extracellular levels of DA, 5-HT, and glutamate in the rat frontal cortex may be explained by agonist activity of 25I-NBOMe at 5-HT_2A_ receptors for which this drug has sub-nanomolar affinity (Rickli et al. 2015). 5-HT_2A_ receptors are highly expressed on glutamatergic pyramidal neurons and GABAergic basket and chandelier cells in layer V of the prefrontal cortex (Halberstadt 2015). The levels of 5-HT_2A_ receptor expression in neuronal cells vary in cortical regions. It is suggested that 50–60% of pyramidal neurons and only 20% of interneurons express 5-HT_2A_ receptors in the frontal cortex (Celada et al. 2013). However, it has been demonstrated that cortical pyramidal glutamatergic cells and GABAergic interneurons also express 5-HT_1A_ receptors (Aznar et al. 2003; Santana et al. 2004). Furthermore, 5-HT_2C_ receptor mRNA and protein are expressed in the frontal cortex, although with relatively weak intensity (Pompeiano et al. 1994). In layer V, these receptors are thought to be expressed postsynaptically in a subset of pyramidal neurons and GABAergic interneurons (Clement et al. 2000; Liu et al. 2007; Pompeiano et al. 1994). Thus, stimulation of 5-HT_2A_ and 5-HT_1A_ receptors can be expected to have mixed effects, with some excitation due to local effects on pyramidal neurons as well as some inhibition due to stimulation of GABAergic interneurons. The available data also suggest that 5-HT_2A/2C_ receptor stimulation by DOI leads to an overall activation on neuronal firing in the frontal cortex (Puig et al. 2003). Hallucinogens may regulate cortical GABA through activation of 5-HT_2A_ receptors located on GABAergic interneurons (Zhou and Hablitz 1999). For instance, DOI administration through a microdialysis probe increased extracellular GABA in the rat prefrontal cortex (Abi-Saab et al. 1999).

25I-NBOMe, besides sub-nanomolar affinity for 5-HT_2A_ receptors, is potent in binding to 5-HT_2C_ receptors and shows weaker but still high affinity for 5-HT_1A_ receptors (Rickli et al. 2015). The observed stimulatory effect of 25I-NBOMe on extracellular glutamate level in our study may be explained by mixed activity of the drug at serotonin receptors and seems to be dependent on the balance between the excitation of pyramidal cells and inhibition of GABAergic interneurons. The balance between excitatory and inhibitory response seems to be related with the 25I-NBOMe dose. The dose-response effect of 25I-NBOMe was *U*-shaped as the low (1 mg/kg) dose was similar in potency to the highest one (10 mg/kg) with respect to increasing glutamate release (Fig. 2a). The middle 25I-NBOMe dose of 3 mg/kg was the weakest in this effect while the dose of 0.3 mg/kg was inactive. Thus, the net effect on glutamate release may depend on the drug level in the brain and activation of different serotonin receptor subpopulations located in various cortical cells by 25I-NBOMe. In contrast to tryptamine hallucinogens, which besides being non-selective agonists of serotonin receptors are substrates for SERT (Nagai et al. 2007; Cozzi et al. 2009; Halberstadt and Geyer 2011), NBOMes are direct agonists of 5-HT_2_ receptors. Therefore, the regulation of cortical glutamate extracellular level by 25I-NBOMe does not need necessarily be dependent on endogenous 5-HT level, as is shown in our study. Fantegrossi et al. (2010) reported biphasic dose-dependent effect of DOI on head-twitch behavior in mice. Those authors suggest that interaction of distinct serotonin receptors generates biphasic nature of the HTR response. It is possible that agonist 5-HT_2A_ receptor activity of 25I-NBOMe at the dose of 1 mg/kg stimulates both glutamate release and head-twitch behavior, whereas the inhibition of both effects observed at the dose of 3 mg/kg may result from a competing agonist activation of 5-HT_2C_ receptors. Wet dog shakes (WDS) produced by NBOMe compounds in Sprague-Dawley rats were suppressed by higher doses of these drugs providing evidence that 5-HT_2C_ receptor stimulation can inhibit 5-HT_2A_-mediated WDS (Elmore et al. 2018; Vickers et al. 2001). Interestingly, increasing the 25I-NBOMe dose to 10 mg/kg resulted in enhancement of extracellular glutamate to the nearly similar level as that produced by the dose of 1 mg/kg. WDS response also increased but was weaker in comparison to the response to 1 mg/kg dose. The reasons for the increase in both effects are not apparent from the in vitro data. The potency of NBOMes at other receptors (adrenergic, dopaminergic) or monoamine transporters is low (Rickli et al. 2015). However, 25I-NBOMe exhibits in vitro affinity for 5-HT_1A_ receptors with *K*_*i*_ = 1800 nM (Rickli et al. 2015). 5-HT_1A_ receptors are expressed on cortical GABAergic interneurons (Aznar et al. 2003; Santana et al. 2004). Hence, activation of 5-HT_1A_ receptors by high concentration of the drug may reveal inhibitory influence of GABA interneurons facilitating effect of 5-HT_2A_ receptors on glutamate release and WDS response.

Cortical pyramidal neurons send long-distance axons to subcortical regions forming loops by which they modulate dopaminergic and serotonergic transmission (Di Matteo et al. 2008; Soiza-Reilly and Commons 2011). 5-HT_2A_ receptors on pyramidal neurons may stimulate directly DA cells in the ventral tegmental area (VTA) or 5-HT cells in the raphe nuclei. This action may be modulated by 5-HT_1A_ or 5-HT_2C_ receptors localized on GABAergic interneurons not only in the frontal cortex but also in the VTA or raphe cells. Thus, GABAergic interneurons provide a link between several classes of serotonin receptors. Concomitant activation the 5-HT_2A_ or 5-HT_2C_ receptors releases GABA which inhibits mesocortical DA cells as well as 5-HT neurons. In addition, activation of 5-HT_1A_ autoreceptors triggers directly the inhibition of serotonin neuron transmission (Di Matteo et al 2008; Soiza-Reilly and Commons 2011).

As observed in our study, activation of serotonin receptors by 25I-NBOMe caused an increase in DA and 5-HT release in the frontal cortex (Fig. 1a, b). The dose-response effect showed an inverted “U” shape with a middle dose of 3 mg/kg being the most potent. Thus, the pattern of the effect exerted by 25I-NBOMe on DA and 5-HT release did not reflect the changes in cortical glutamate release produced by the drug. It is likely that a weaker stimulation of descending pathways by this dose exerted a weaker impact on GABAergic interneurons to inhibit DA and 5-HT cells in the VTA or raphe nuclei. Subsequently, more neurotransmitters were released from neuronal terminals in the frontal cortex. This effect may be modulated by direct action of 25I-NBOMe via 5-HT_2A_ receptors on 5-HT cells or 5-HT_2A_ and 5-HT_2C_ receptors on GABAergic interneurons (Di Matteo et al 2008). At higher dose of 10 mg/kg by direct action via 5-HT_1A_ autoreceptors 25I-NBOMe may suppress raphe cells firing causing the observed decrease in 5-HT release from cortical nerve terminals (Haigler and Aghajanian 1973). The inverted “U” shape manner of the neurotransmitter release was mimicked by the measured 5-HT and 5-HIAA content in the frontal cortex (Fig. 3b). Similarly, direct modulation of VTA dopaminergic neurons through activation of 5-HT_2A_ receptors by 25I-NBOMe may affect DA release from cortical terminals (Cornea-Hébert et al. 1999; Nocjar et al. 2002; Pehek et al. 2001). Some 5-HT_2C_ receptors situated on GABAergic interneurons in the VTA may suppress triggering of DA release by higher doses of the drug (Di Matteo et al. 2008; Nocjar et al. 2002; Pehek et al. 2001). 25I-NBOMe did not enhance significantly the contents of DA and its metabolites and only a tendency towards the increase was observed (Fig. 3a). Thus, the pattern of changes in DA release does not reflect the changes in the contents of DA and its metabolites. It may be hypothesized that the lack of parallelism between changes in release and tissue DA content may result from different regulation of neurotransmitter synthesis and release. DA synthesis may depend more on excitatory projections from the cortex reaching cell bodies in the VTA while synaptic DA release may be modulated by local tonic influences via serotonin receptors on neuronal terminals.

25I-NBOMe displayed hallucinogenic activity as shown by the number of head shakes measured during 4 h after drug administration. Interestingly, the number of shakes followed the pattern of changes in glutamate release with the middle dose of 3 mg/kg being the weakest in this test (Fig. 2b). This is the evidence that hallucinogenic activity of 25I-NBOMe seems to be associated with glutamate release in the cortex and may be mediated via 5-HT_2A_ receptors. Our data are the first to connect hallucinogenic-like behavior in rodents with glutamate release in the frontal cortex after administration of this sub-class of hallucinogens. So far, there are only two studies showing an increase in cortical glutamate release by LSD and DOM (Muschamp et al. 2004) or DOI (Scruggs et al. 2003). Still, HTR was shown in mice (Halberstadt and Geyer 2014) and in Sprague-Dawley rats (Elmore et al. 2018).

Taken together, the results of our study expand our knowledge on a new very potent group of hallucinogens represented by 25I-NBOMe. The obtained data show that 25I-NBOMe affects DA, 5-HT, and glutamatergic neurotransmission. The observed hallucinogenic activity of 25I-NBOMe may be associated with glutamate release in the frontal cortex.

## Electronic supplementary material

ESM 1 (DOCX 94 kb)
