# Supplementary material for: Hallucinogen-Like Action of the Novel Designer Drug 25I-NBOMe and Its Effect on Cortical Neurotransmitters in Rats
Source: Neurotox Res. 2019 Apr 15;36(1):91–100. doi: 10.1007/s12640-019-00033-x (PMC6570696; doi:10.1007/s12640-019-00033-x)
Supplement: Supplementary file 1 — (DOCX 94 kb) [file 12640_2019_33_MOESM1_ESM.docx]

**Table 1** Tissue contents of DA, DOPAC, HVA, 5-HT, and 5-HIAA in the frontal cortex measured 45 min after administration of 25I-NBOMe

| Treatment (mg/kg) | DA | DOPAC | HVA | 5-HT | 5-HIAA |
| --- | --- | --- | --- | --- | --- |
| Frontal cortex pg/mg wt ± SEM (n) | | | | | |
| Control | 471 ± 69 (6) | 136 ± 18 (6) | 150 ± 23 (6) | 589 ± 27 (6) | 297 ± 22 (6) |
| 25I-NBOMe 0.3 | 536 ± 102 (6) | 122 ± 23 (6) | 144 ± 26 (6) | 650 ± 90 (6)^ab^ | 290 ± 18 (6)^a^ |
| 25I-NBOMe 1 | 540 ± 43 (6) | 146 ± 20 (6) | 180 ± 20 (6) | 782 ± 43 (6)**^aa^ | 316 ± 12 (6)^aa^ |
| 25I-NBOMe 3 | 511 ± 70 (6) | 143 ± 29 (6) | 175 ± 33 (6) | 929 ± 16 (6)** | 361 ± 8.9 (6)** |
| 25I-NBOMe 10 | 600 ± 49 (6) | 168 ± 21 (6) | 194 ± 29 (6) | 846 ± 34 (6)**^ab^ | 353 ± 13 (6)*^b^ |

* *P* < 0.05, ** *P* < 0.01 versus control group; a *P* < 0.05, aa *P* < 0.01 versus 25I-NBOMe 3; b *P* < 0.05, bb *P* < 0.01 versus 25I-NBOMe 1 (one-way ANOVA and Tukey’s post hoc test)
